# Excessive Exposure to Secondhand Tobacco Smoke among Hospitality Workers in Kyrgyzstan

**DOI:** 10.3390/ijerph7030966

**Published:** 2010-03-10

**Authors:** Denis Vinnikov, Nurlan Brimkulov, Shahida Shahrir, Patrick Breysse, Ana Navas-Acien

**Affiliations:** 1 Public Association “Lung Health”, Togolok Moldo Street, 1, Bishkek, Kyrgyzstan; E-Mail: brimkulov@list.ru; 2 Department of Environmental Health Sciences and Institute for Global Tobacco Control, John Hopkins University, Bloomberg School of Public Health, Baltimore, MD, USA; E-Mails: sshahrir@jhsph.edu (S.S.); anavas@jhsph.edu (A.N.-A.); pbreysse@jhsph.edu (P.B.)

**Keywords:** passive smoking, public health policy, workplace

## Abstract

The aim of this study was to assess the levels of secondhand smoke (SHS) exposure of men and women in public places in Kyrgyzstan. This cross-sectional study involved 10 bars and restaurants in Bishkek the capital city of Kyrgyzstan. Smoking was allowed in all establishments. Median (interquartile range) air nicotine concentrations were 6.82 (2.89, 8.86) μg/m^3^. Employees were asked about their smoking history and exposure to SHS at work. Employees were exposed to SHS for mean (SD) 13.5 (3.6) hours a day and 5.8 (1.4) days a week. Women were exposed to more hours of SHS at work compared to men. Hospitality workers are exposed to excessive amounts of SHS from customers. Legislation to ban smoking in public places including bars and restaurants is urgently needed to protect workers and patrons from the harmful effects of SHS.

## 1. Introduction

Secondhand tobacco smoke (SHS) has been recognized as a major cause of death and morbidity, and has been classified as a carcinogen by the International Agency for Research on Cancer (IARC) and the US Environmental Protection Agency [1,2]. Evidence has accumulated, mainly during the last 20 years, that SHS causes lung cancer, nasal sinus cancer, asthma and heart disease, among other conditions [3]. Exposure can occur anywhere people spend their time – at work, at home and in public places. The 2002 study of SHS-related deaths in 28 Western European countries estimated 79,000 SHS-related deaths a year [4].

SHS exposure may be much higher among hospitality industry workers compared to other population groups [5]. First, SHS exposure levels in bars and restaurants are substantially higher compared to other public places and work places [6,7]. Second, bar and restaurant workers have high pulmonary ventilation rates that result in increased pulmonary and systemic exposure to SHS and greater respiratory symptoms [8]. When smoke-free legislation covering the hospitality industry is applied, SHS exposure in this group of workers is dramatically reduced [9]. Countries that have adopted smoke-free legislation show immediate reductions in SHS levels and improvements in the respiratory health of the hospitality industry workers [10–12].

Exposure to SHS in public places depends on the presence and enforcement of smoking ban legislation. The international standard promoting smoke-free policies around the world is the World Health Organization Framework Convention on Tobacco Control (FCTC), the first global public health treaty. Article eight of the FCTC underlines the need to implement comprehensive smoke-free policies that protect all workers from the harmful effects of tobacco smoke. This treaty mandates governments of all ratifying countries implement effective legislative measures to protect citizens from deadly effects of tobacco smoke in all public places and workplaces, including transportation and hospitality establishments.

In Kyrgyzstan, an incomplete legislation that restricts and in some cases prohibits smoking in public places was enacted at the end of 2006. No studies have evaluated SHS exposure in public places in Kyrgyzstan. Due to partial regulation, bar employees may be exposed to even higher levels of SHS compared to the general public. In Kyrgyzstan, the majority of workers in the hospitality industry are female, making women more likely to experience SHS exposure at work. The aim of this study was to assess air nicotine levels in a sample of bars and restaurants in Bishkek, the capital city of Kyrgyzstan, and to evaluate SHS exposure in the men and women who work in these places.

## 2. Design and methods

### 2.1. Study Design

This project was part of a multi-country cross-sectional study to assess SHS exposure in bars and nightclubs around the world. The study protocol was approved by the Johns Hopkins Bloomberg School of Public Health Institutional Review Board and the Ethical Committee on Biomedical Research in the Ministry of Health of Kyrgyz Republic. Fieldwork was carried out in Bishkek, the capital of Kyrgyzstan, in August and September of 2008.

### 2.2. Bars and Restaurants

Establishments invited to participate in this study were located in Bishkek neighborhoods with a high density of public places where people spend time or gather socially. We invited a total of 120 bars to take part in this study, and obtained consent from only 10 owners/managers (response rate 8%). All venues allowed smoking. The minimal eligibility requirement for an establishment to be included in the study was that at least one employee was a non-smoker and willing to participate. In each location, the bar owner completed an interview-based questionnaire. They were asked to describe the smoking status of employees and patrons, indoor smoking policy, whether cigarettes were sold inside the bar and how—such as the presence of tobacco advertizing and promotion, as well as general characteristics of the establishment.

Two secondhand smoke monitors were left in each venue for seven days—one in a smoking room, the other in a non-smoking room (in one of the establishments only). The monitors were checked during the busiest time of the third day to ensure placement and to note the number of people present. The location of each sampler was recorded onto a diagram. The total room volume and the presence of air conditioners/ventilators were noted in rooms where monitors were hung.

To ensure quality control, 10% of the monitors were placed next to the main sampler as duplicate samples. Another 10% of the monitors were assigned blanks used to calculate the blank-corrected nicotine concentrations and limit of detection.

### 2.3. Participants

Five employees per venue were recruited, including one active smoker and four non-current smokers. If the establishment employed fewer than four non-smoking workers, all employees were invited to participate. Participation was anonymous, voluntary and required informed consent. A total of 33 employees consented to participate; they answered a 30-minute interviewer-based questionnaire and provided a hair sample. The questionnaire collected the following information: sociodemographic characteristics, work characteristics and experience, past/present smoking status, and exposure to SHS at work and other places, including the smoking habits of their household members. We also requested self-reported information about their respiratory health and possible SHS-related symptoms. The final section of the questionnaire was on general views and attitudes to smoking bans in public places.

Hair samples were collected at the end of the interview for nicotine measurement. Approximately 30−50 strands of hair were cut near the hair root from at the back of the scalp. The minimum length of hair from each study subject was 3 cm.

### 2.4. Nicotine Measurement

Airborne nicotine samples were collected using a passive sampler. Samples were analyzed using gas chromatography with nitrogen detection. The airborne concentration of nicotine was computed by dividing the amount of nicotine collected by each filter (μg) by the volume of air sampled (m^3^). The latter is equal to the total of sampling time in minutes multiplied by the flow rate (25 mL/min). More detail on this method can be found in [6,13].

Hair nicotine was measured by gas chromatography mass spectrometry according to Kim *et al*. [14]. The hair concentration of nicotine was calculated by dividing the amount of nicotine collected in each hair sample (ng) by the volume of hair sampled (mg). For quality control, 10% hair samples were measured in duplicate.

All analyses were performed at the Exposure Assessment Facility of the Institute for Global Tobacco Control, the John Hopkins Bloomberg School of Public Health.

### 2.5. Statistical Analysis

Sampling and questionnaire data were processed using Microsoft Access and analyzed using Stata version 9.0. Descriptive statistics included frequencies, percentages, means, medians and interquartile ranges. Non-parametric tests (Mann-Whitney, Wilcoxon) were used for comparisons of hair nicotine concentrations by participant characteristics and SHS exposure at work, home and in other environments. In multivariate analyses, air and hair nicotine were the main outcome variables.

## 3. Results

### 3.1. Area Sampling Results

Participating venues were in business for a mean (SD) of 4.2 (3.2) years and operated for mean (SD) of 14.6 (3.8) hours a day. Other characteristics of the venues are presented in Table 1.

**Figure 1.**
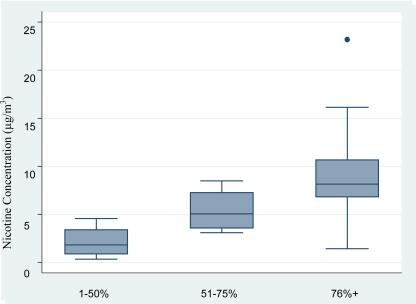
Air nicotine concentration based on percentage of smoking customers.

Smoking was allowed in all of restaurants and bars participating in this study. Only one establishment had a non-smoking room, however, it was connected to a room that allowed smoking through an open doorway. Smoking was permitted in 95% of the rooms where the air monitors were placed. None of the venues had air nicotine concentrations below the limit of detection, including the non-smoking room. Air nicotine levels ranged from 0.34 to 23.19 μg/m^3^. Air nicotine levels increased in a dose-response manner with the proportion of customers who smoke as reported by the establishment owner/manager. Median nicotine concentrations in bars with a greater percentage of smoking customers were higher than those with fewer smoking customers (Table 2 and Figure 1). The limit of detection (LOD) of nicotine in air was 0.003 μg/m^3^ for a seven-day air sample.

Even the single non-smoking room detected noteworthy levels of air nicotine (8.02 μg/m^3^), though half the concentration found in the adjacent smoking room (16.1 μg/m^3^). The lowest concentration of air nicotine was seen in a smoking venue corresponding to a restaurant reported to have fewer than 25% smoking customers, the least proportion of smokers.

### 3.2. Participants Results

Employees (N = 33) surveyed in this study were mainly women (N = 27), and the mean (SD) age of responders was 25.8 (6.9) years (from 18 to 52). They were mainly high school graduates (60.6%), but 39.4% had higher education. 39.4% worked as waiters, 30.3% as managers, 12.1% as bartenders, 6.1% as owners, and 12.1% as other. One-third smoked daily during the last year, and cigarettes were almost the only product they used for smoking (Table 3). Women were more likely not to have tried a single puff ever (55.5% *versus* 16.7%).

Study participants reported being exposed to SHS at work for mean (SD) 5.8 (1.4) days a week and for mean (SD) 13.5 (3.6) hours a day. Women were exposed to SHS at work for more hours compared to men [14.1(3.0) *versus* 10.5(4.7) h]. 78.8% of employees self-reported tobacco smell in their clothes when they returned home.

Study participants also reported significant exposure to SHS at home. All except one participant lived with at least one other adult (from one to four), and 51.5% reported having at least one smoker at home (10 participants with one smoker, seven participants with two smokers). More women were exposed to SHS from a person they lived with than men (59.3% of women lived with a smoker *versus* 16.6% of men). In households where the study participant lived with a smoker, 30% would smoke indoors, smoking on average 9.4 (SD 7.2) cigarettes. In households where there were two smoking members, the second smoker would smoke on average 10.1 (mean 7.4) cigarettes indoors. Only in two of the households in which a smoker lived, smoking indoors was not allowed at all.

Overall, support for smoke-free public places in general was fairly small, only 18%. However, 82% indicated that they would prefer to work in a smoke-free environment. Women were more likely to support totally smoke-free venues compared to men (22.2% *versus* 0%).

### 3.3. Nicotine in Hair Sampling Results

The limit of detection (LOD) of nicotine in hair was 0.08 ng/mg. The median (IQR) hair nicotine concentration in smokers was 2.48 ng/mg (0.08−52.82) compared to 0.84 ng/mg (0.08−3.87) in non-smokers (p < 0.01, Table 4). Hair nicotine concentration had a moderate positive correlation with total number of cigarettes smoked by household members (r = 0.44, p < 0.01).

## 4. Discussion and Conclusions

Secondhand smoke is an important public health concern that causes premature death and disease and impacts the quality of life, in particular, for hospitality industry employees exposed to SHS for many hours. In this study, we confirmed that exposure to SHS was very common in Kyrgyzstan, and must be urgently reduced. Some steps have been taken to reduce SHS exposure in public places, but comprehensive legislation that covers the hospitality industry is still needed.

Data obtained from this study support establishing a full smoking ban in all public places around the country. Airborne nicotine concentrations evaluated in Kyrgyzstan are comparable to SHS assessment in hospitality venues of other European countries and much higher than levels measured in Latin America [6,17,18]. The median nicotine concentration in European restaurants (2.09 μg/m^3^) and Latin American restaurants (median 1.24 μg/m^3^) and bars (median 3.65 μg/m^3^) were somewhat lower to the levels that we found in Kyrgyzstan bars and restaurants. Data are also consistent with studies done in other locations lacking smoke-free policies in public places [5].

In 2006 legislation was passed in Kyrgyzstan which aimed to protect the general public from the harmful effects of tobacco smoke. This law required owners of socializing venues to reserve at least 50% of their public space for nonsmokers. Legislation allowing separated areas inadequately protects employees from SHS exposure. While Kyrgyzstan has been attributed to a group of countries “making progress with smoke-free policies” [15], much work remains to be done in Kyrgyzstan to protect the hospitality employee from very high exposure levels to secondhand tobacco smoke.

Fieldwork in the present study was carried out in early autumn 2008 and it clearly demonstrated that tobacco control efforts at that time were largely ineffective. People working in this sector are still subjected to high levels of smoke, as reflected in their self-reported questionnaires, air and hair nicotine levels. Another concern was the large number of respiratory complaints and the general poor health of hospitality employees, many of whom suffered from chronic respiratory conditions. Consequently, smoke-free legislation is the simplest remedy for the morbidities associated with the modifiable etiological factor, tobacco smoke.

Data obtained in this study support establishing full smoking bans in all public places and workplaces around the country. 82% of employees stated they would prefer to work in a smoke-free environment. Being exposed up to 14 hours of SHS is unacceptable, and public health efforts are urgently needed to dramatically reduce SHS exposure in these venues. It has been shown that a comprehensive policy is the only measure that substantially lowers exposure to SHS with positive benefits for cardiovascular and respiratory health [11,12].

SHS in the workplace is undoubtedly a problem; more so when compounded with exposure in other places like in the home. Women are found to be at greater risk of SHS exposure when compared to men both at work and at home. They spent more hours per day at work and were more likely to live with at least one smoker compared to men. In addition to asking employees about their exposure to secondhand smoke at home and at work, we also measured hair nicotine concentrations.

Our findings support the need for urgent protection of hospitality industry employees from SHS in the workplace. However, the study had certain limitations, which must be taken into account when comparing these data with other settings. Firstly, we could cooperate only with those establishments that agreed to be included in the study. The volunteer nature of participation coupled with the low response rate could potentially bias the study findings. In particular, it is possible those bars accepting to participate had lower SHS exposure levels compared to non-respondent bars. Secondly, there were very few men in the study, but this was the general trend in the hospitality industry. Also, the sample size was small, and the study was only carried out in Bishkek. Lastly, the project was done in autumn when a large number of those who frequent these socializing venues prefer to stay outdoors, weather permitting. We may expect exposure to SHS to be much higher in winter. Overall, given the study limitations, the situation of SHS exposure in Kyrgyzstan may be much worse.

In summary, we found levels of exposure to SHS both in socializing venues and at home to be high in participants in this study conducted in Kyrgyzstan. Hospitality workers were exposed to excessive amounts of SHS from customers, working many hours a day in places where there were little to no smoking restrictions. These findings support the need to enforce making all public places totally smoke-free to protect their employees from harmful effects of tobacco smoke.

## Figures and Tables

**Table 1.** Description of the venues participating in the study.

Venue	Type	Indoor area (m^2^)	Maximum occupancy	No. employees	Smoking allowed	Cigarettes sold	Tobacco ads	Mean air nicotine (μg/m^3^)
1	Karaoke bar	500	60	9	Yes	Yes	No	6.82
2	Pizza place	400	25	10	Yes	No	No	8.85
3	Pizza place	230	90	25	Yes	Yes	No	12.08
4	Billiard club	200	70	4	Yes	Yes	No	15.73
5	Night club	500	120	25	Yes	Yes	No	10.38
6	Restaurant	80	70	20	Yes	Yes	No	0.86
7	Restaurant	250	130	60	Yes	Yes	Yes	3.41
8	Cafe	135	70	15	Yes	Yes	Yes	3.59
9	Cafe	70	80	8	Yes	Yes	No	2.06
10	Cafe	360	120	27	Yes	Yes	Yes	7.27
All*	**N = 10**	**293 (161)**	**83.5 (32.3)**	**20.3 (16.1)**	**100%**	**90%**	**30%**	**7.11**

* Mean (SD) or percentage.

**Table 2.** Air nicotine concentrations overall and by self-reported % customers who smoke.

			Air nicotine (μg/m^3^)			
	No. venues	No. monitors	P25	P50	P75	P90
All venues	10	20	2.89	6.82	8.86	13.83
1–50% customers smoke	2	4	0.86	1.82	3.41	4.57
51–75% customers smoke	2	4	3.59	5.06	7.27	8.49
76% or more customers smoke	6	12	6.82	8.15	10.66	16.14

**Table 3.** Demographic and smoking profiles of hospitality industry employees.

	Total (N = 33)	Smokers (N = 13)	Non-smokers (N = 20)
% women	81.8	69.2	90.0
Age, years	25.8 (6.9)	24.5 (4.8)	26.8 (8.0)
Work duration, years	1.7 (0.9)	1.6 (0.8)	1.8 (1.1)
Work days a week	5.3 (1.3)	5.2 (1.3)	5.4 (1.4)
Work hours a day	12.5 (2.8)	12.1 (2.6)	12.7 (2.9)
Age of smoking initiation	17.8 (4.4)	17.8 (4.4)	n/a
Number of cigarettes smoked per day	9.5 (4.9)	9.5 (4.9)	n/a

Data shown as means (standard deviation).

**Table 4.** Level of nicotine in the hair of bar employees.

	Hair nicotine (μg/mg)			
	N	P50	P75	P90
Current smokers	11	0.90	2.48	12.47
Non-smokers	22	0.14	0.84	1.89
